# *E. coli* genetically modified for purine nucleobase release promotes butyrate generation and colonic wound healing during DSS insult

**DOI:** 10.1080/19490976.2025.2490211

**Published:** 2025-04-17

**Authors:** J. Scott Lee, Daniel J. Kao, Corey S. Worledge, Zachary F. Villamaria, Ruth X. Wang, Nichole M. Welch, Rachael E. Kostelecky, Sean P. Colgan

**Affiliations:** a Department of Medicine, Mucosal Inflammation Program, University of Colorado Anschutz Medical Campus; b Department of Medicine, Rocky Mountain Veterans Association, Aurora, CO, USA

**Keywords:** Microbiota-derived metabolites, butyrate, hypoxanthine, purines, energy metabolism, epithelial barrier, wound healing, proliferation, host-microbe interactions, next-generation probiotic

## Abstract

The gut microbiota transforms energy stored as undigestible carbohydrates into a remarkable number of metabolites that fuel intestinal bacterial communities and the host tissue. Colonic epithelial cells at the microbiota–host interface depend upon such microbiota-derived metabolites (MDMs) to satisfy their energy requisite. Microbial dysbiosis eliciting MDM loss contributes to barrier dysfunction and mucosal disease. Recent work has identified a role for microbiota-sourced purines (MSPs), notably hypoxanthine, as an MDM salvaged by the colonic epithelium for nucleotide biogenesis and energy balance. Here, we investigated the role of MSPs in mice during disease-modeled colonic energetic stress using a strain of *E. coli* genetically modified for enhanced purine nucleobase release (*E. coli* Mutant). *E. coli* Mutant colonization protected against DSS-induced tissue damage and permeability while promoting proliferation for wound healing. Metabolite and metagenomic analyses suggested a colonic butyrate-purine nucleobase metabolic axis, wherein the *E. coli* Mutant provided purine substrate for Clostridia butyrate production and host purine salvage, altogether supplying the host substrate for efficient nucleotide biogenesis and energy balance.

## GRAPHICAL ABSTRACT

**Figure:**
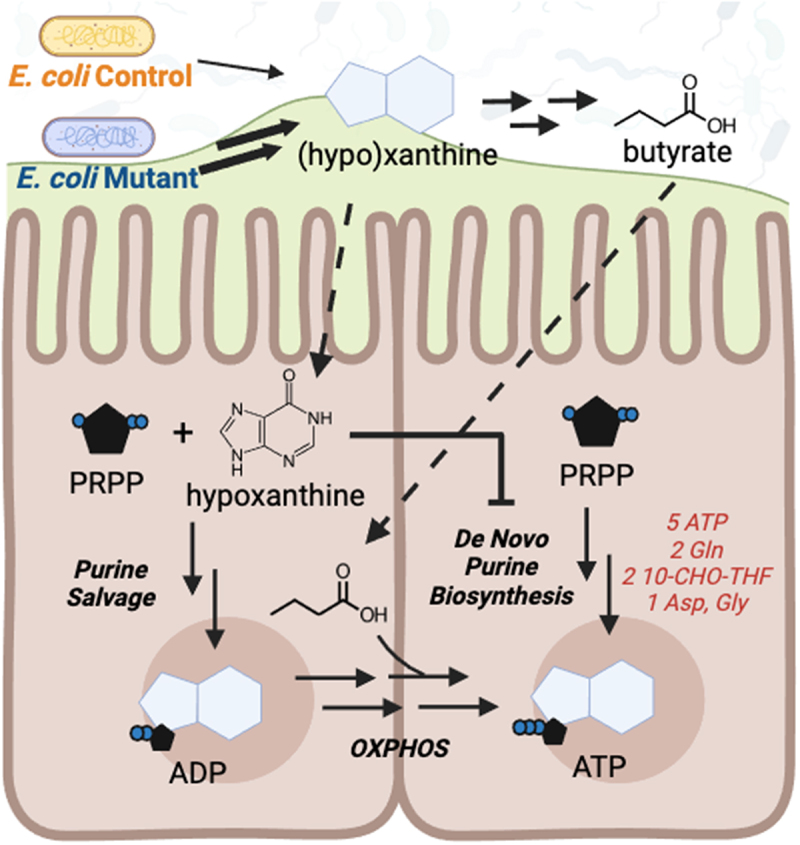

## Introduction

Trillions of microorganisms make up the gut microbiota that mutualistically reside in the human colon during health. This microbiota works as a bioreactor that transforms the energy stored as otherwise indigestible carbohydrates into substrates that fuel intestinal bacterial communities and the host.^1,2^ Because gut microbes participate in complex cross-feeding relationships where the metabolic end product of one strain is the preferred energy source of another, a deficiency in one component of this cross-feeding relationship can resonate across the microbiota and induce global shifts in composition and function.^3,4^ This deficiency may then deprive host organ systems of requisite metabolites,^5^ critically so with regard to the colonic epithelium, which has evolved a fundamental dependency on microbiota-derived metabolites (MDMs) for energy procurement. Microbiota-derived butyrate is well known as the primary fuel source of colonocytes.^6,7^ A steep concentration gradient exists between luminal and systemic butyrate concentrations with 95% absorbed by colonocytes.^8–10^ Luminal butyrate is absorbed by apical transporters and serves as a primary substrate for β-oxidation to form acetyl-CoA, which can be used for fatty acid, sterol biosynthesis, or drive oxidative phosphorylation to regenerate ATP from ADP.^11–14^ It has long been known that colonocytes during ulcerative colitis (UC) are marked by a metabolic defect of diminished butyrate oxidation with enhanced glucose and glutamine oxidation.^15^ In general, fecal butyrate levels are considered diminished in inflammatory bowel disease (IBD) patients,^16–19^ concomitant with decreases in butyrate-producing species^1,20,21^ and the butyryl-CoA:acetate-CoA-transferase gene (the primary mechanism of butyrate synthesis)^22,23^ in the microbiota. This shift in butyrate availability and colonocyte metabolism from butyrate to glucose and glutamine utilization is maladaptive because of the decreased relative energetic yield. It is thought that a consequence of this change in MDM provision and host metabolism contributes to chronic intestinal disease due to a state of energy deficiency impeding wound healing – the “starved gut” hypothesis.^15,24,25^

**Figure 1.**
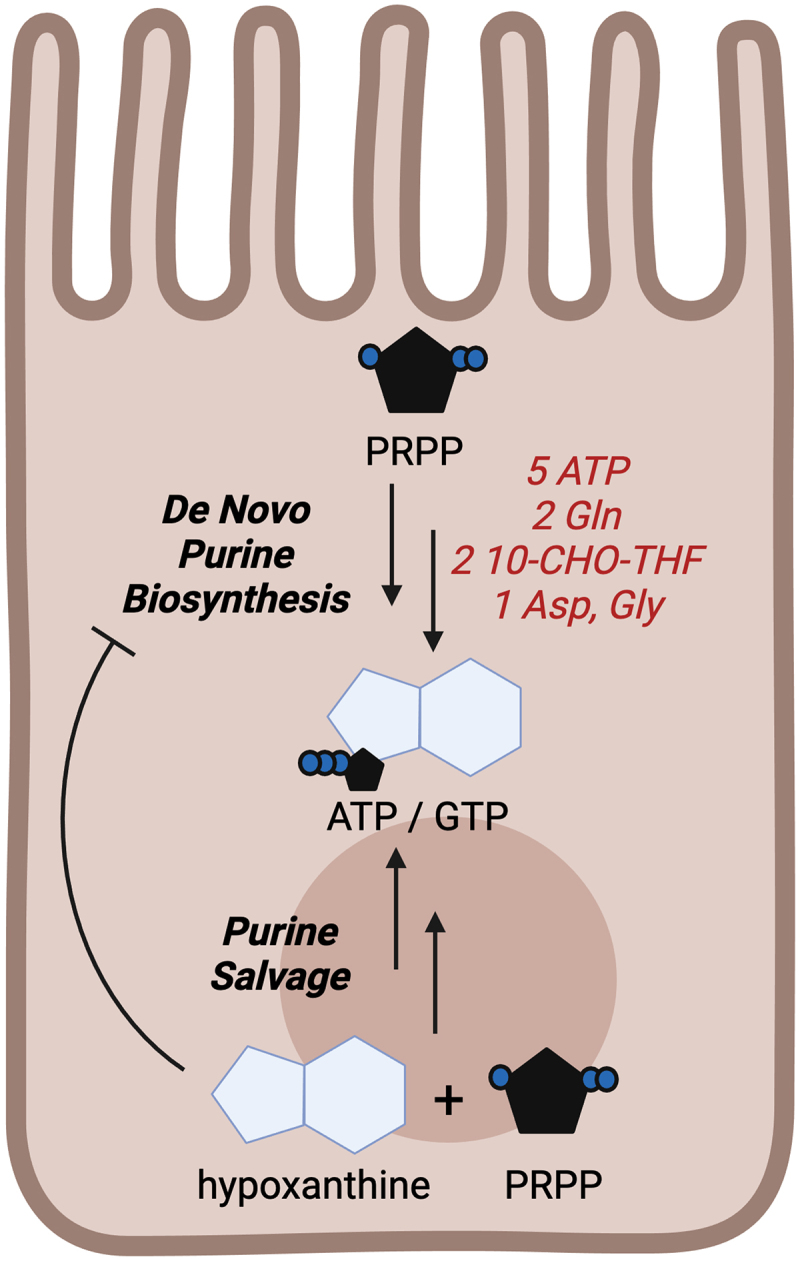
Purine salvage spares energy and substrate relative to *de novo* biosynthesis. Graphical representation highlighting the energy and substrate requirements for *de novo* purine biosynthesis relative to purine salvage. PRPP, phosphoribosyl pyrophosphate; Gln, glutamine; 10-CHO-THF, 10-formyltetrahydrofolate; Asp, aspartate; Gly, glycine; ATP adenosine triphosphate; GTP, guanosine triphosphate.

The dependency on MDMs for colonic energy procurement is well exemplified in germ-free (GF) mice, where tissue extracts show ~55% of the available high-energy phosphates (ATP, PCr) relative to conventional-raised CR counterparts at baseline.^26^ A substantial proportion of this microbiota-derived energy is utilized by intestinal epithelial cells at the interface of microbiota–host interactions. Development and maintenance of the apical junctional complex in addition to generation and secretion of highly glycosylated mucin proteins for barrier is very substrate- and energy-demanding,^26,27^ but essential for mutualistic microbiota–host interactions and gut homeostasis. The lack of microbiota-sourced purines (MSPs) for salvage by colonocytes as a significant contributor to a starved gut is gaining appreciation, and for good reason as intestinal epithelial cells preferentially salvage purines *in lieu* of the energy- and substrate-demanding *de novo* biosynthesis of purines (Figure 1).^28–30^

Accumulation of the purine nucleobase hypoxanthine (Hpx) commonly associates with stress and disease such as hypoxia,^31^ colorectal cancer,^32^ dementia and Alzheimer’s disease,^33^ and multiple sclerosis.^34^ This Hpx accumulation indicates a stress-induced shift of decreased ATP production and regeneration, and/or increased ATP utilization, resulting in purine nucleotide degradation.^27^ Although Hpx increases during stress and disease, exogenous Hpx supply and accumulation promotes energy balance, wound healing, cytoskeletal capability, and tight junctional formation and function *in vitro*.^27^ This contribution of Hpx supply to the colonic epithelium was recapitulated *in vivo* through streptomycin-induced MSP depletion and reconstitution via Hpx supplementation or re-colonization with purine nucleobase-releasing bacteria. These studies demonstrated that the murine colonic epithelium is dependent upon MSPs for energy balance, survival, proliferation, and mucus barrier integrity during DSS insult.^26^ Other recent work also highlights a significant role for Hpx supply in intestinal health, where an Hpx-starved epithelium due to increased *Lachnospiraceae* consumption was identified as a potential mechanism underlying irritable bowel syndrome (IBS).^35^ Depletion of fecal purines also associates with ulcerative colitis (UC), as indicated by an untargeted metabolomic screen of fecal metabolites showing 36% Hpx (*p* = 0.003) and 33% xanthine (Xan, *p* < 0.004) decreases (https://www.metabolomicsworkbench.org/Project ID PR000677).^36^ Furthermore, fecal microbiota transplantation (FMT) was shown to increase Hpx and Xan in promotion of sustained remission.^37^

Presented herein are studies employing a genetically modified *E. coli* enriched in Hpx and Xan release to further delineate the role of these MSPs in gut health and wound healing. This genetically modified *E. coli* (*E. coli* Mutant) was used to reconstitute purine-depleted mice prior to induction of DSS insult.^26^ This work further supports that MSPs are fundamental to colonic energy metabolism, wound healing, and barrier function. Furthermore, we identify a previously undescribed convergence of Hpx and butyrate metabolism. To this end, we explore the role of MSPs as a cross-feeding substrate in which purines supplied by the *E. coli* Mutant support butyrate production by Clostridia. Altogether, this work identifies a fundamental purine-butyrate axis in microbe–microbe and microbe–host interactions necessary to maintain function in health and during disease.

## Results

### The genetically modified *E.* *coli* is enriched in hypoxanthine and xanthine distribution

**Figure 2.**
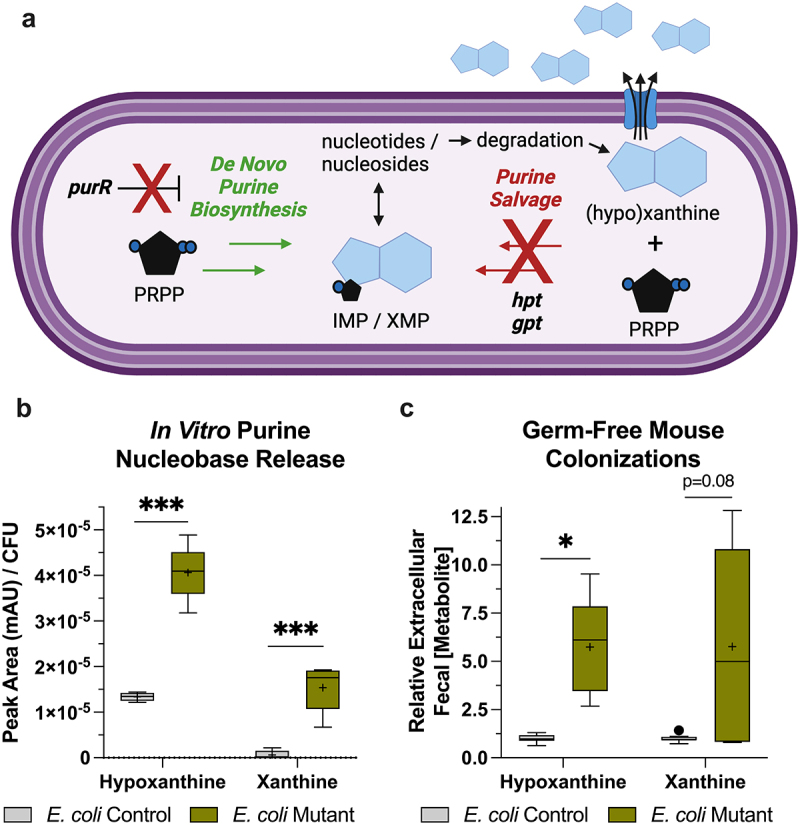
The *E. coli* mutant is enriched in hypoxanthine and xanthine release. (a) Graphical representation of the how the *purR, hpt, and gpt* mutations in the *E. coli* mutant activate *de novo* purine biosynthesis and inhibit purine salvage, leading to nucleobase accumulation and release. (b) Extracellular hypoxanthine and xanthine released by *E. coli* control and mutant *in vitro* (*n* = 6). (c) Relative extracellular hypoxanthine and xanthine levels in fecal extracts from *E. coli* control and mutant germ-free mouse monocolonizations (*n* = 5–8). Data are represented as Tukey box and whiskers with median designated by the line and mean the +. * indicates *p* < 0.05, ***p indicates <0.001; PRPP, phosphoribosyl pyrophosphate; IMP, inosine monophosphate; XMP, xanthosine monophosphate.

As a starting point, we generated an *E. coli* Mutant that inactivates the primary repressor of *de novo* purine biosynthesis genes (purR) to stimulate the *de novo* purine biosynthetic pathway, as well as hypoxanthine phosphoribosyltransferase (hpt) and xanthine-guanine phosphoribosyltransferase (gpt) to inhibit the purine nucleobase salvage pathway and altogether promote purine nucleobase accumulation and release (Figure 2(a)). To determine if the mutations induced purine nucleobase release, *E. coli* Control and Mutant cultures were grown in a purine-deficient, minimal media and analyzed for extracellular purine nucleobases. The *E. coli* Mutant showed substantially more Hpx and Xan release (*p* < 0.001), with no other purine nucleobases observed (Figure 2(b)). This Hpx and Xan release was also observed in GF mice monocolonized with the *E. coli* Control or Mutant, with Mutant extracellular fecal extracts showing an ~ 6-fold increase in steady-state hypoxanthine (Hpx, *p* = 0.01) and xanthine (Xan, *p* = 0.08) relative to *E. coli* Control colonization (Figure 2c).

### The *E.* *coli* mutant increases colonic (Hypo)xanthine and ATP

**Figure 3.**
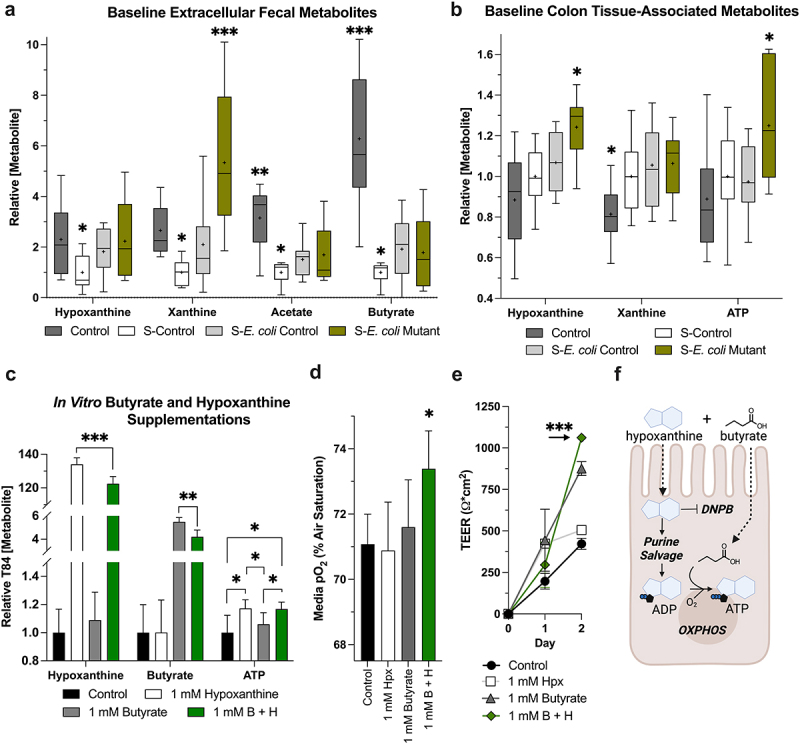
Streptomycin- and colonization-induced metabolite shifts at baseline and *in vitro* assessment of hypoxanthine and butyrate metabolic synergy. (a) Relative extracellular fecal metabolite levels in control, streptomycin-treated control, and streptomycin-treated and *E. coli*-colonized mice (*n* = 9–16). (b) Corresponding colon-tissue associated purine and ATP shifts (*n* = 9–10). (c) Relative metabolite levels in control, hypoxanthine-supplemented, butyrate-supplemented, and co-supplemented T84 cells (*n* = 5–6). (d) Media oxygen levels as a metric of T84 oxygen consumption (*n* = 6). (e) Transepithelial resistance measurements during the initial phase of barrier formation in treated T84 cells (*n* = 6). (f) Graphical representation depicting the synergistic metabolism of hypoxanthine and butyrate for ATP generation. A and B data are represented as Tukey box and whiskers with median designated by the line and mean the +. C – E data are represented as mean ± SD. * indicates *p* < 0.05, ** indicates *p* < 0.01, *** indicates *p* < 0.001; significance indicators without lines showing comparison designate that value as significant compared to all other groups. S, streptomycin; Hpx, hypoxanthine; ADP, adenosine diphosphate; ATP, adenosine triphosphate; B + H, butyrate and hypoxanthine co-supplementation; TEER, transepithelial resistance, DNPB, *de novo* purine biosynthesis; OXPHOS, oxidative phosphorylation.

Streptomycin-induced colonic purine depletion in conventionally raised mice was employed to assess the influence of purine reconstitution by colonization with streptomycin-resistant *E. coli* Control and Mutant. The dysbiosis incited by streptomycin (S-Control) reduced steady-state extracellular fecal Hpx (*p* < 0.03) and Xan (*p* < 0.001), concomitant with acetate and butyrate (*p* < 0.001, Figure 3(a)). We presume that these Hpx and butyrate decreases impose a state of energy metabolic deficiency on the colonic epithelium. Colonization with *E. coli* Control (S-*E. coli* Control) appeared to restore homeostatic fecal Hpx and Xan levels (*p* < 0.02), with partial acetate (*p* = 0.03) and butyrate (*p* = 0.009) restoration. The enriched release of purine nucleobases by the *E. coli* Mutant (S-*E. coli* Mutant) shifted the fecal and colon tissue metabolic profile relative to *E. coli* Control. Extracellular fecal Hpx (*p* = 0.009) also appeared restored with partial acetate (*p* < 0.02) and butyrate (*p* = 0.009) recovery, but with Xan greatly increased even compared to Control (*p* < 0.001). Interestingly, *E. coli* Mutant colonization also induced a significant colon-tissue associated Hpx increase (*p* < 0.02), as though Hpx partitioned to the tissue and Xan to the lumen, with an associating tissue ATP increase (*p* < 0.05, Figure 3(b)). Trace amounts of urate, guanine, and adenine were detected throughout the analyses but were not determined due to their substantially lower levels compared to Hpx and Xan.

### Butyrate and hypoxanthine synergize as metabolic substrates

To assess the relative contribution of Hpx and butyrate to ATP levels and a potential Hpx-butyrate metabolic axis, overnight butyrate, Hpx, and butyrate + hpx (B + H) supplementation experiments were performed on T84 intestinal epithelial model cells. All supplementations significantly elevated intracellular levels of the corresponding metabolite(s) (*p* < 0.001). Supplementation with Hpx alone proved responsible for ATP genesis in this model, but, interestingly, butyrate and Hpx were synergistically metabolized in the co-treatment (*p* < 0.01). In this, more butyrate and Hpx were being consumed in the B + H group relative to the butyrate and Hpx-only groups (Figure 3(c)). Given the increased butyrate consumption observed in the co-treatment, oxygen consumption analyses were performed. Following overnight incubation, B + H-treated cells were found consuming less oxygen than all other groups, indicative of decreased mitochondrial activity (*p* = 0.04, Figure 3(d)). A following experiment monitoring the influence of the supplementations on the rate of tight junction formation after plating (sub-confluent state) was performed. All treatment groups exhibited similar final higher junctional resistances significantly greater than control T84 cells (*p* < 0.05, data not shown), but the metabolic synergy in the B + H treatment functionally manifested as an increased junctional formation rate (*p* < 0.001), likely through supporting the high level of cytoskeletal activity necessary for polarization and junction formation (Figure 3(e)).^27,38,39^ Altogether, these *in vitro* results provide initial evidence that butyrate and Hpx are synergistically metabolized to efficiently drive energy production and cellular function (Figure 3(f)).

### The *E.* *coli* mutant functionally enriches the microbiota in butyrate production

**Figure 4.**
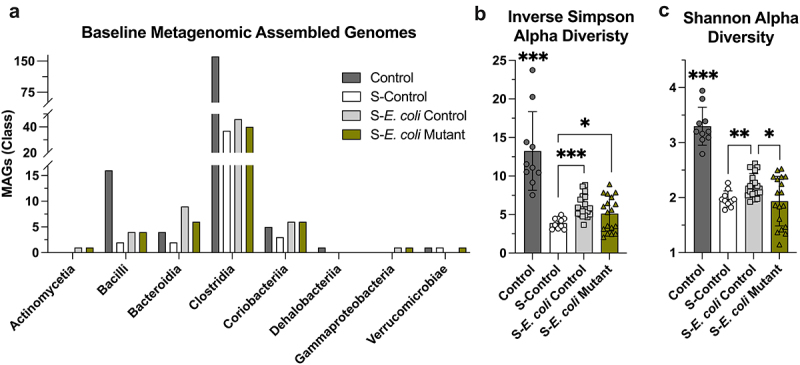
Streptomycin reduces Clostridia and Bacilli metagenomic-assembled genomes. (a) The number of metagenomic-assembled genomes (MAGs) recovered from each microbiota. MAGs are grouped by class (*n* =10–18). (b) MAGs inverse Simpson alpha diversity (*n* =10–18). (c) MAGs Shannon alpha diversity (*n* =10–18). B and C data are represented as mean ± SD. * indicates *p* < 0.05, ** indicates *p* < 0.01, *** indicates *p* < 0.001; significance indicators without lines showing comparison designate that value as significant compared to all other groups. S, streptomycin.

Shotgun metagenomic sequencing of fecal samples and construction of metagenomic-assembled genomes (MAGs) was performed to assess the influence of MSP nucleobases on microbiota composition and function. This work was performed using Anvi’o.^40–42^ Genomes were reconstructed from the shotgun metagenomic sequencing (metagenome-assembled genomes, MAGs) and annotated with functions from the KOfam HMM database of KEGG orthologs.^43,44^ Taxonomic estimates in Anvi’o currently uses single-copy core genes (SCGs) found in Genome Taxonomy Database (GTDB) genomes (Supplementary Data – Taxonomy).^45^ Streptomycin treatment was shown to greatly decrease the number of MAGs generated from Bacilli and Clostridia classes (Figure 4(a)). This influence also manifested as significantly decreased alpha diversity in both the Inverse Simpson and Shannon indices (*p* < 0.001, Figures 4(b,c)). The colonizations appeared to minimally influence the number of Bacilli and Clostridia MAGs produced relative to S-Control, with some recovery in Inverse Simpson alpha diversity (*p* < 0.04) and slight recovery in Shannon alpha diversity with *E. coli* Control colonization (*p* < 0.002).

**Figure 5.**
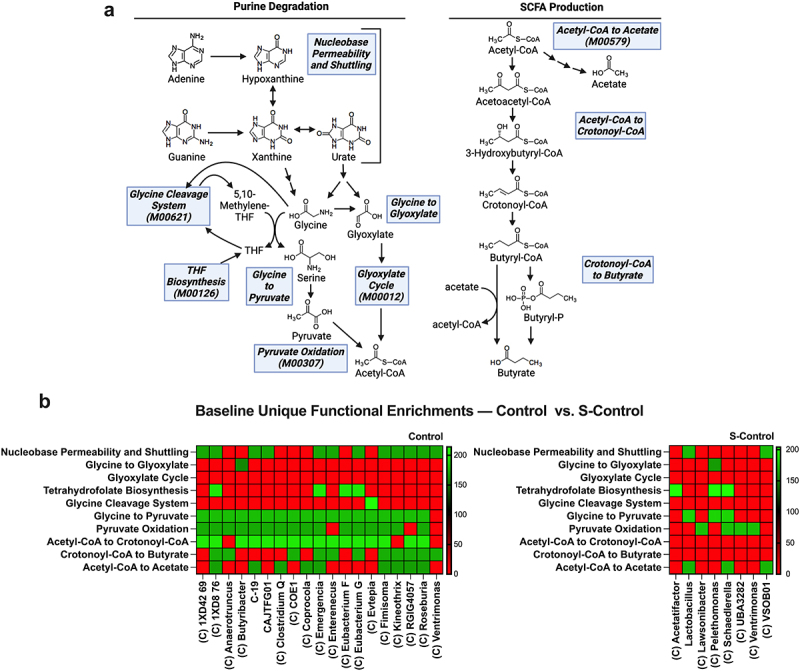
Streptomycin depletes the microbiota of butyrate-producing metabolic pathways. (a) Metabolic pathway summary of microbial purine degradation for acetate and butyrate production highlighting the metabolic pathway modules used to search for genomic functional enrichment. Existing KEGG modules are indicated by the KEGG accession number. (b) Control vs. S-Control baseline functional enrichment comparison. Uniquely enriched genus metagenomic-assembled genome groups are shown, except control groups not exhibiting butyrate-producing enrichments. C indicates the genus group is Clostridia. S, streptomycin.

To investigate the functional capabilities of the microbiotas, genomic functional enrichment comparisons of KEGG modules (metabolic pathways) and custom user-defined modules relevant to purine nucleobase transport and conversion of nucleobases to Xan and urate, their degradation to acetyl-CoA, and SCFA generation from acetyl-CoA were performed amongst the MAGs in groups at the genus taxonomic level (MAGs grouped as per genus with each group compared to all other groups) (Figure 5(a)).^46–48^ Enrichments of q < 0.05 were considered significant (Supplementary Data – Functional Enrichments). Separate modules for acetyl-CoA to crotonoyl-CoA and crotonoyl-CoA to butyrate were constructed to specifically analyze for butyrate production from acetyl-CoA, as crotonoyl-CoA can also be generated from lysine, glutamate, and succinate.^49,50^ Microbial purine degradation pathways begin with Xan and urate.^49–53^ The Xan to glycine degradation pathway currently does not have KEGG orthologs in the database to construct metabolic pathway modules for analysis, while modules designed to probe degradation from urate to glycine and/or glyoxylate did not show any unique enrichments across the groups. Analysis of unique, butyrate-producing functionality (defined by a unique group enrichment in the Acetyl-CoA to Crotonoyl-CoA and/or Crotonoyl-CoA to Butyrate modules) between Control and streptomycin-treated control (S-Control) revealed streptomycin treated mice as deficient in microbiota butyrate biosynthetic capability (Figure 5(b)). This coincided with decreases in the Nucleobase Permeability and Shuttling, Glycine to Pyruvate, Pyruvate Oxidation, and Acetyl-CoA to Acetate modules, indicating that SCFA generation in this model appears specifically linked to acetyl-CoA biosynthesis from glycine via serine.

**Figure 6.**
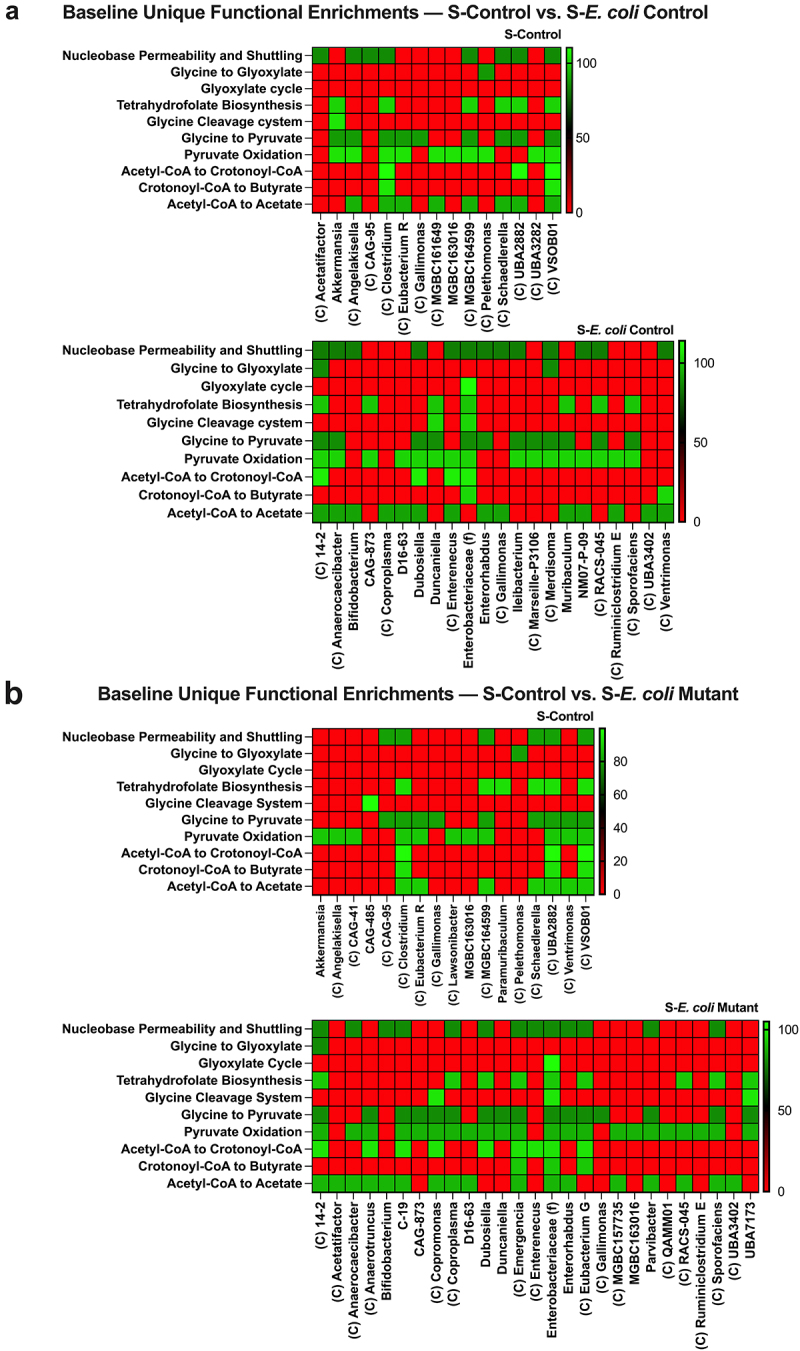
The *E. coli* mutant reconstitutes a streptomycin-depleted microbiota with more butyrate-producing genomic enrichments than the *E. coli* control. (a) S-control vs. S-*E. coli* control baseline functional enrichment comparison. (b) S-control vs. S-*E. coli* mutant baseline functional enrichment comparison. Uniquely enriched genus metagenomic-assembled genome groups are shown. C indicates the genus group is Clostridia, f indicates the group resolved to the family level. S, streptomycin.

**Figure 7.**
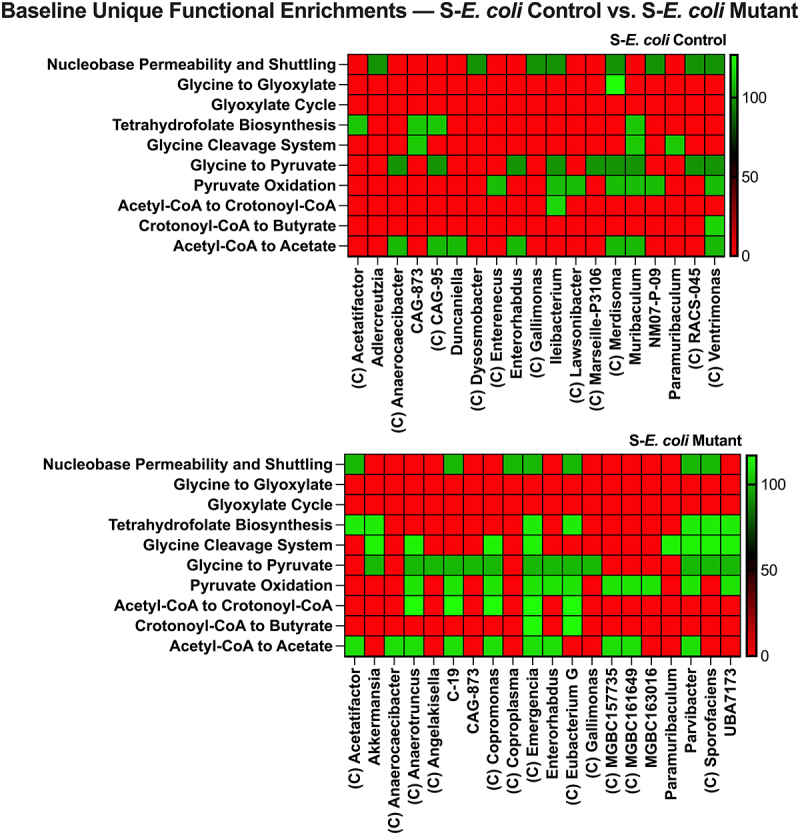
*E. coli* Mutant-colonization enriches microbiota butyrate-producing pathways relative to *E. coli* control. (a) S-*E. coli* control vs. S-*E. coli* mutant baseline functional enrichment comparison. Uniquely enriched genus metagenomic-assembled genome groups are shown. C indicates the genus group is Clostridia, f indicates the group resolved to the family level. S, streptomycin.

Colonization with the *E. coli* Control increased the number of enriched Nucleobase Permeability and Shuttling groups but only produced two more unique butyrate-producing enrichments relative to S-Control (Figure 6(a)), whereas *E. coli* Mutant colonization tripled the number of unique butyrate pathway enrichments (Figure 6(b)). This difference was further highlighted in a S-*E. coli* Control and S-*E. coli* Mutant comparison showing how the increased purine release by the mutant enriches glycine degradation via serine and pyruvate oxidation in addition to several butyrate pathway enrichments (Figure 7). Notably, most of these butyrate-associated functional shifts in the microbiotas involved Clostridia, this is intriguing as Clostridia is well known for purine utilization and SCFA production.^51,54–57^

### *E.* *coli* mutant colonization protects against colonic insult and promotes epithelial recovery

**Figure 8.**
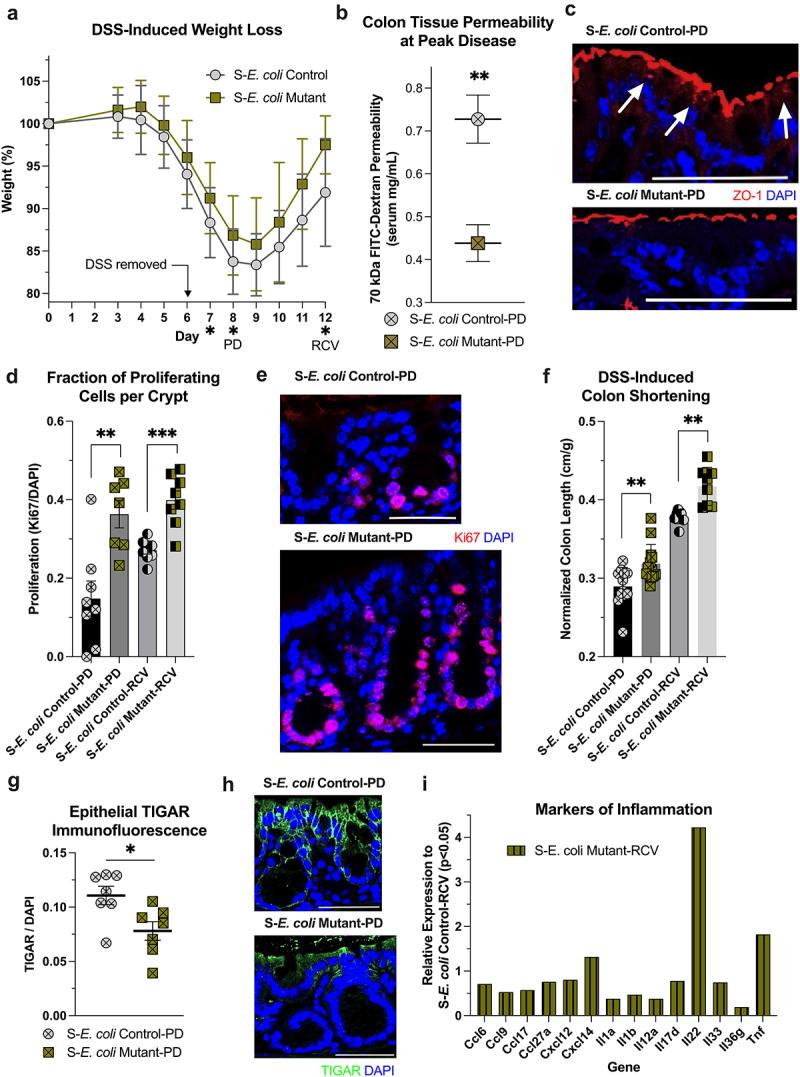
The *E. coli* mutant protects against DSS insult and promotes wound healing. (a) Weight curve time course of the DSS insult and recovery. Peak disease (PD, day 8) and recovery (RCV, day 12) time points are highlighted. * indicates days showing significant weight differences (*p* < 0.05, *n* = 8–18). (b) Serum fitc-dextran levels of mice given oral gavage with a 70 kDa marker to assess colonic tissue permeability (*n* = 4). (c) Murine colonic ZO-1 immunofluorescent analyses of *E. coli* control- and mutant-colonized mice at peak disease. Arrows indicate regions of exceptional ZO-1 dyslocalization. Bar indicates 50 μm. (d) Fraction of proliferating cells per crypt in *E. coli* control- and mutant-colonized mice at peak disease and during recovery (*n* = 7–8). (e) Representative murine colonic Ki67 immunofluorescent images from *E. coli* control- and mutant-colonized mice at peak disease. Bar indicates 50 μm. (f) Colon length measurements normalized to mouse initial weight as a metric of disease severity (*n* = 8–13). (g) TIGAR immunofluorescent analyses of experimental groups during peak disease. Immunofluorescent analyses have an *n* = 31–35 quantitated images from 7 mice. (h) Representative murine colonic TIGAR immunofluorescent images from *E. coli* control- and mutant-colonized mice at peak disease. Bar indicates 50 μm. (i) *E. coli* mutant-colonized colon tissue chemokine and cytokine expression relative to *E. coli* control (*p* < 0.05). A, B, and F data are represented as mean ± SD, D and G as mean ± SEM, I as mean. * indicates *p* < 0.05, ** indicates *p* < 0.01, *** indicates *p* < 0.001. S, streptomycin; PD, peak disease; RCV, recovery.

The influence of MSPs in a disease context was investigated using *E. coli* Control and *E. coli* Mutant-colonized mice subjected to DSS colonic insult. In previous work, it was found that streptomycin attenuates DSS-induced disease severity, with streptomycin-treated, *E. coli* Control-colonized mice showing nearly complete protection against weight loss and colon shortening.^26^ As such, the dose and course were tailored to incite significant disease in the *E. coli* Control in order to assess the influence of the *E. coli* Mutant. Treating colonized mice with 4–4.5% DSS in drinking water for 6 days was determined to induce a diseased state. Mice colonized with the *E. coli* Mutant were protected against the insult, with significantly attenuated weight loss on days 7 (*p* = 0.04), 8 (*p* < 0.04), and 12 (*p* = 0.05, Figure 8(a)). Additionally, a colonic tissue permeability assessment on day 8 during peak disease (PD) showed substantially decreased FITC-Dextran permeability in *E. coli* Mutant-colonized mice compared to Control-colonized mice (*p* = 0.007, Figure 8(b)). Epithelial ZO-1 immunofluorescence (IF) quantification (ZO-1/DAPI) showed no significant difference between the colonizations, but *E. coli* Mutant-colonized mice displayed normal ZO-1 junctional localization, in contrast to the dysfunctional localization observed in the *E. coli* Control-colonized mice, suggesting improved cytoskeletal function (Figure 8(c)).^58,59^ Proliferation was estimated by Ki67 IF at peak disease and during recovery (RCV, day 12) to gauge wound healing. *E. coli* Mutant-colonized mice showed a significantly increased fraction of crypt proliferating cells at both time points, whereas the S-*E. coli* Control proliferative response appeared delayed (Figures 8d,e). Accordingly, S-*E. coli* Mutant mice had significantly longer colon lengths at both time points (Figure 8(f)).

Given the potential synergistic metabolic relationship between Hpx and butyrate presented above, TP53-induced glycolysis and apoptosis regulator (TIGAR) IF levels were also analyzed at peak disease. TIGAR can reflect stress and is relevant to purine metabolism through regulating ribose-5-phosphate and thus phosphoribosyl pyrophosphate (PRPP) production for purine *de novo* biosynthesis or salvage (Figure 1), inhibits proliferation, and responds to oxidative stress for reactive oxygen species (ROS) mediation.^60–62^ Epithelial TIGAR levels were significantly elevated in S-*E. coli* Control-PD (*p* < 0.02, Figure 8(g)). TIGAR levels generally increased in proximity to the lumen, but the induction in *E. coli* Control mice extended deeper into the lower, proliferative crypt region (Figure 8(h)). Histological scoring of hematoxylin and eosin-stained (H&E) colon tissue sections revealed no major differences in inflammation between the colonizations at either time points, suggesting that streptomycin diminishes inflammation and shifts the model more toward DSS-induced tissue damage. Nonetheless, bulk tissue RNA sequencing was performed at the recovery time point to assess markers of inflammation. In this, colon tissue from S-*E. coli* Mutant-RCV appeared to exhibit significantly lower expression levels of pro-inflammatory factors (*p* < 0.05) with a notable 4.2-fold (*p* = 0.02) IL-22 increase (Figure 8i, Supplementary Data – RNAseq Differential Expression), a cytokine known to promote epithelial regeneration and barrier function.^63^

### The *E.* *coli* mutant promotes Clostridia while increasing fecal xanthine and butyrate during recovery

**Figure 9.**
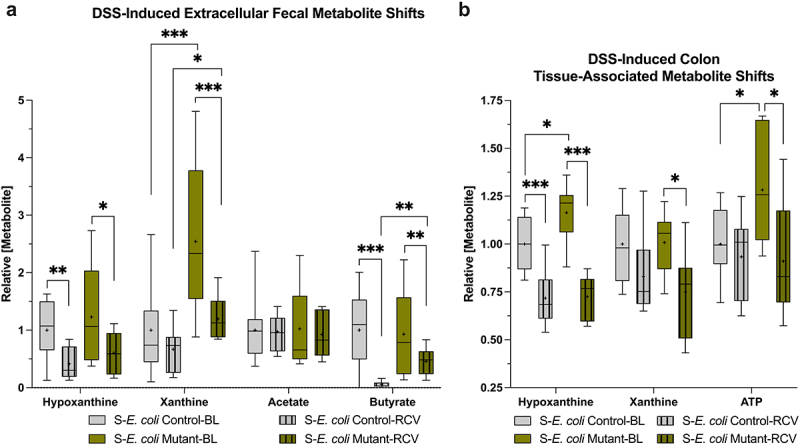
Baseline to recovery metabolite shifts in *E. coli* control- and mutant-colonized mice. (a) Relative extracellular fecal metabolite levels at baseline and recovery in streptomycin-treated and *E. coli*-colonized mice (*n* = 7–16). (b) Corresponding colon-tissue associated purine and ATP shifts (*n* = 7–10). Data are represented as Tukey box and whiskers with median designated by the line and mean the +. * indicates *p* < 0.05, ** indicates *p* < 0.01, *** indicates *p* < 0.001. BL, baseline; RCV, recovery; ATP, adenosine triphosphate.

**Figure 10.**
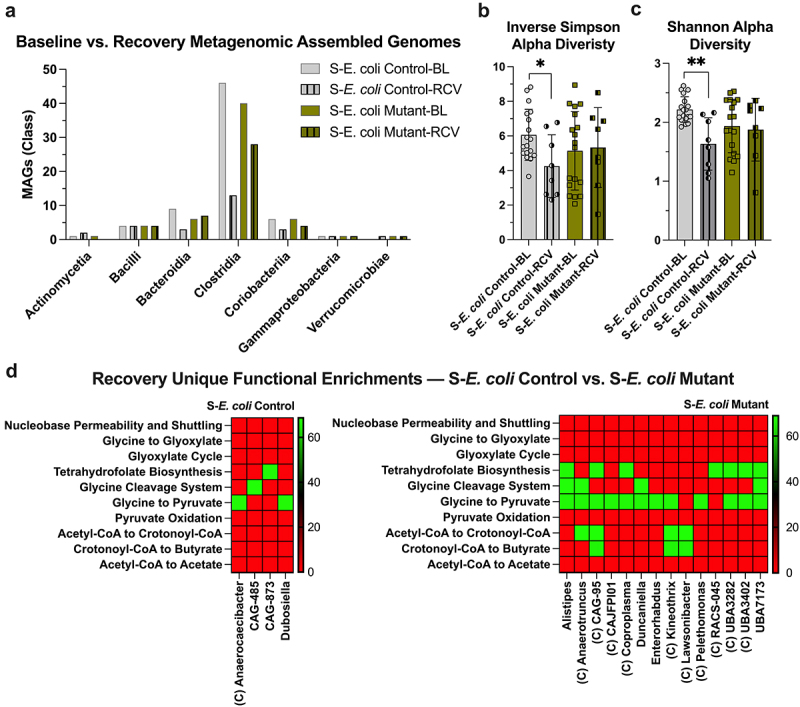
The *E. coli* mutant protects against DSS-induced Clostridia depletion. (a) The number of metagenomic-assembled genomes (MAGs) constructed from baseline and recovery microbiotas in streptomycin-treated and *E. coli*-colonized mice. MAGs are grouped by class (*n* = 8–18). (b) MAGs inverse Simpson alpha diversity (*n* = 8–18). (c) MAGs Shannon alpha diversity (*n* = 8–18). (d) S-*E. coli* control vs. S-*E. coli* mutant recovery functional enrichment comparison (q < 0.1). Uniquely enriched genus metagenomic-assembled genome groups are shown. C indicates the genus group is Clostridia. B and C data are represented as mean ± SD. * indicates *p* < 0.05, ** indicates *p* < 0.01. S, streptomycin; BL, baseline; RCV, recovery.

The insult incurred by DSS substantially shifted the murine fecal and colonic tissue metabolite profiles. During recovery, mirroring ulcerative colitis fecal untargeted metabolite analyses,^36^ luminal Hpx and Xan significantly decreased relative to baseline levels, but S-*E. coli* Mutant-RCV still maintained a significantly higher xanthine level (Figure 9(a)). No differences in steady-state extracellular fecal acetate was observed, but S-*E. coli* Control-RCV mice showed near-complete butyrate depletion while the *E. coli* Mutant supported an increased butyrate level (*p* < 0.005). Colon tissue-associated Hpx significantly decreased from baseline during recovery with both colonizations, in addition to S-*E. coli* Mutant-RCV appearing to fully utilize the baseline Hpx and ATP reserve (Figure 9(b)). The DSS insult primarily shifted microbiota composition by decreasing Clostridia, but the *E. coli* Mutant appeared to support double the Clostridia MAGs than the *E. coli* Control (Figure 10(a)). This difference was also reflected by only *E. coli* Control colonizations showing decreased Inverse Simpson (*p* < 0.03) and Shannon (*p* = 0.007) alpha diversities (Figure 10(b,c)). Functional enrichment comparisons between the colonizations during recovery were also performed. No enrichments were observed with q < 0.05, suggesting the butyrate response may be more defined by MSP nucleobase supply as a limiting substrate, rather than microbiota functional capability. Enrichments of q < 0.1 showed *E. coli* Mutant colonization as generally enriching the Glycine to Pyruvate module with four unique butyrate-producing enrichments, while S-*E. coli* Control-RCV showed no unique butyrate-producing enrichments (Figure 10(d)).

## Discussion

In this work, we introduce a murine model of streptomycin-induced metabolic stress. Consistent with previous work,^7^ the loss of microbiota-derived metabolites (MDMs), particularly butyrate, significantly reshapes the metabolic landscape of the colon. These MDMs include a reservoir of MSP nucleobases in the colonic lumen, primarily Hpx and Xan, of which their depletion associate with the ulcerative colitic disease state. Given our previous observations and the current work,^26^ we propose the energetic stress experienced by the host from decreased Hpx availability is due in large part by activation of *de novo* purine biosynthesis (DNPB) and resultant sequestering of energy and nutrients, given that purinosomes (DNPB enzyme clusters) localize to the mitochondria for this purpose.^64–66^ Hpx supplementation alone, not butyrate, increased ATP in T84 cells, demonstrating the large energy requirement of DNPB. In biochemical terms, DNPB consumes 5 ATP molecules relative to salvage of one Hpx molecule and even low micromolar concentrations of exogenous Hpx demonstrate significant DNPB inhibition.^67^ In this work, the increase in MSP nucleobase supply provided by the *E. coli* Mutant associated with decreased weight loss, decreased tissue permeability with homeostatic-like ZO-1 junctional organization, and increased proliferation with decreased colon shortening as indicators of attenuated disease severity in response to DSS insult.

It is reasonable that a primary driver of protection is the availability of salvageable MSP nucleobases providing the host substrate for nucleotide generation and proliferation (DNA duplication) and sparing the epithelium from the energy- and substrate-demanding DNPB. The induction of TIGAR lower into the proliferative crypt region in *E. coli* Control-colonized mice at peak disease may indicate increased purinosome activity in support of this hypothesis. It is demonstrated that ROS is a significant TIGAR inducer, as TIGAR functions to upregulate glutathione regeneration for ROS remediation.^60,68^ Consequently, TIGAR induction is tied to oxidative stress through mitochondrial ROS generation.^69^ As TIGAR also works to suppress the cell cycle through regulating the RB-E2F1 complex,^70^ a lack of purine substrate for nucleotide biogenesis triggering DNPB, oxidative stress, and then TIGAR induction may represent a purine supply-associated regulatory mechanism. In times of insufficient exogenous purine supply eliciting purinosome-driven oxidative stress, this mechanism could work to simultaneously inhibit proliferation, remediate ROS, and increase PRPP production for DNPB initiation.

Streptomycin is known to deplete intestinal SCFAs by decreasing Clostridia.^57^ This observation was confirmed in this work, where streptomycin greatly decreased the number of Clostridia MAGs. This is significant regarding MSP nucleobases as Clostridia are also known for high purine utilization, notably Xan, and streptomycin concomitantly depletes the microbiota of these purines.^51,54–57^ Given that purine degradation provides a source for acetyl-CoA, and butyrate generation from acetyl-CoA is the primary pathway utilized by the microbiota,^49^ it is likely that streptomycin-induced butyrate depletion is in some part the result of decreased purine substrate. In support of this idea, the Nucleobase Permeability and Shuttling module enrichment generally appeared to associate with butyrate pathway enrichments, notably in the S-Control comparisons. Furthermore, although analysis of Xan to glycine degradation is not currently possible due to lacking the associating orthologs in the KEGG database and the constructed urate to glycine degradation pathway analyses revealed no enrichments across the experiments, this degradation can be inferred through associated genomic enrichments in the Glycine to Pyruvate and Pyruvate Oxidation modules. These enrichments also signal a preferred route of purine degradation to acetyl-CoA through glycine and serine. Interestingly, neither colonization restored the streptomycin-induced Clostridia loss but rather appeared to shape the function of this class through MSP-dependent butyrate-producing pathway enrichments.

It is reasonable to expect *E. coli* Mutant colonization to increase luminal butyrate levels at baseline, which was not observed. It is postulated that more butyrate may indeed be produced but is more quickly consumed due to the increased colon tissue-associated Hpx and synergistic Hpx and butyrate utilization, as observed *in vitro*. Intriguingly, luminal butyrate was near entirely depleted with *E. coli* Control colonization during recovery, presumably due to both a decrease in microbial butyrate genesis and increased host consumption to regenerate the ATP required to drive DNPB. The rationale for monitoring acetate is due to butyrate production typically coinciding with net acetate consumption by well-known and abundant Clostridia butyrate producers such as *Faecalibacterium prausnitzii*, *Eubacterium rectale*, and *Anaerobutyricum (Eubacterium) hallii*,^22,23,50,71^ establishing acetate as a potential limiting substrate in butyrate production. Acetate does not appear to be a limiting substrate in this model, as luminal levels did not change from baseline to the recovery state and the Acetyl-CoA to Acetate module appeared widely distributed. If there is an increased microbial butyrate production component to the S-*E. coli* Mutant-RCV butyrate increase, it may primarily stem from increased mutant MSP nucleobase release providing substrate for degradation, as no genomic enrichments were observed with a q < 0.05. Enrichments with a q < 0.1 showed *E. coli* Mutant colonization as generally enriching the Glycine to Pyruvate module with four unique Clostridia butyrate-producing enrichments compared to S-*E. coli* Control-RCV showing none. These genomic enrichments resolve to *Anaerotruncus sp000403395*, *CAG-95 sp910587295*, *Kineothrix sp011958945*, and *Lawsonibacter sp910588635* MAGs. The *Anaerotruncus* MAG also showed enrichment with *E. coli* Mutant colonization at baseline, along with the Control-BL microbiota, supporting its presence during recovery. Accordingly, *Anaerotruncus* are identified as butyrate-producing mucin degraders,^72,73^ CAG-95 are predicted butyrate producers that strongly correlate with host health,^74^ *Kineothrix* are butyrate producers shown to positively impact the gut by attenuating metabolic dysfunction-associated steatotic liver disease (MASLD),^75,76^ and *Lawsonibacter* also demonstrate butyrate production and, intriguingly, strongly associate with coffee consumption.^77,78^

Next-generation probiotics will likely be comprised of microbial communities chosen for specific activities and genetically modified microorganisms designed to locally deliver the necessary metabolites for wound healing and disease remission.^79–82^ Genetically engineering probiotic strains to enrich bacteria in therapeutic activities offers a means to address both microbiota and host processes for the reinstatement of homeostatic microbe–microbe and microbiota–host relationships. Inherent to wound healing is sufficient energy input to drive proliferation and barrier restitution, with reinstatement of the microbe–microbe interactions necessary for microbiota-derived metabolite production to support disease remission. The present work suggests that butyrate and Hpx are synergistically consumed for efficient nucleotide biogenesis and energy balance, with MSP promoting microbiota butyrate production. Taken together, MSPs have a fundamental role in gut homeostasis and wound healing with enrichment in purine release representing a promising next-generation probiotic.

## Methods

### Resource availability

#### Lead contact

Further information and requests for resources and reagents should be directed to and fulfilled by the lead contact, J. Scott Lee (joseph.s.lee@cuanschutz.edu).

#### Materials availability

The availability of the *E. coli* Control and Mutant K12 strains used in this study is restricted due to MTA.

#### Data availability

Shotgun metagenomic sequencing data are deposited at the NCBI Sequence Read Archive (SRA) under BioProject ID PRJNA1202651. Any additional information required to reanalyze the data reported in this paper is available from the lead contact upon request.

### Experimental model details

#### Vertebrate animal use

The University of Colorado Anschutz Medical Campus (AMC) animal management program is accredited by the American Association for the Accreditation of Laboratory Animal Care (AAALAC) and meets the National Institutes of Health standards as set forth in the Guide for the Care and Use of Laboratory Animals (DHHS Publication No. (NIH) 85–23). The institution also accepts as mandatory the PHS Policy on Human Care and Use of Laboratory Animals by Awardee Institutions and NIH Principles for the Utilization and Care of Vertebrate Animals Used in Testing, Research and Training.

#### Murine K12 colonizations

##### Bacterial preparation for oral gavage

A glycerol stock of the streptomycin-resistant K12 strains used for the colonization experiments were streaked on LB agarose plates containing streptomycin (50 µg/mL) and grown at 37°C overnight. A single colony was used to inoculate 2 mL of streptomycin-containing LB broth, and the culture grown overnight at 37°C with shaking. A 1.5 mL aliquot of the culture was centrifuged at 3000 g for 5 min to pellet the bacteria, the supernatant removed, and the bacteria then resuspended in 1.5 ml of PBS in preparation for oral gavage. A 100 uL gavage of the suspension is ~ 10^8^ CFUs.

##### Germ-free mouse monocolonization

Germ-free mice were provided by the University of Colorado AMC gnotobiotic core. Female 7–8-week-old C57Bl6 gnotobiotic mice were administered 100 µL of the K12 bacterial preparation by oral gavage and allowed 1 week for colonization and complete turnover of the colonic epithelium. Fecal pellets were then collected, flash frozen, and stored at −80°C until analysis.

##### Conventionally raised mouse purine depletion and K12 reconstitution

Female 8-week-old C57BL/6J mice were provided by the University of Colorado AMC animal facility. Mice from different cages were equally distributed among the experimental groups and a piece of bedding from each initial cage added to the group cages to facilitate normalization of microbiotas. Mice receiving streptomycin were administered the antibiotic in the drinking water (5 g/L), and those being colonized were done so the next day by administration of 100 µL of the K12 bacterial preparation by oral gavage. All mice were then equilibrated with the treatments for 1 week to allow for microbiota equilibration and complete turnover of the colonic epithelium. Mice receiving streptomycin were maintained on the antibiotic throughout all experimentation. Mice submitted to dextran sodium sulfate (DSS) insult were administered 4–4.5% DSS in drinking water for 6 days and sacrificed on day 8 for “peak disease” and day 12 for “recovery” analyses. In previous work, K12 colonization was verified to maintain throughout the DSS treatment.^26^ A piece of colon tissue encompassing the most distal fecal pellet was excised and fixed in methacarn (60% absolute methanol, 30% chloroform, 10% glacial acetic acid, v/v) for histology and immunofluorescence analyses.^83^ A distal piece of colon tissue (~15 mg) was quickly placed in a pre-weighed 1.7 mL centrifuge tube and weighed, 500 µL of ice-cold 80% methanol added, flash frozen by immersion in liquid nitrogen, and stored at −80°C until processed for HPLC metabolite analysis. Fecal pellets were also flash-frozen and similarly stored until analyses.

##### Colonic permeability

Mice were administered 100 µL of 100 mg/mL 70-kDa FITC-dextran (Sigma-Aldrich) by oral gavage and blood collected after 4 h. Serum was isolated via Microvette 500 Z Gel.

### Method details

#### T84 cell culture

Cells were cultured in 95% air with 5% CO2 at 37°C in standard media made of DMEM:F-12 supplemented with 10% calf serum, 1% penicillin/streptomycin, and 1% GlutaMAX™ (ThermoFisher Scientific).

#### E. coli *control and mutant purine nucleobase release*

A glycerol stock of the streptomycin-resistant K12 strains used for the colonization experiments were streaked on LB agarose plates containing streptomycin (50 µg/mL) and grown at 37°C overnight. A single colony was used to inoculate 5 mL of streptomycin-containing M9 with 0.4% glucose (M9+G), and the culture grown overnight at 37°C with shaking. A subculture was then started by inoculating 20 mL of M9+G with 2 mL of the overnight culture and grown at 37°C with shaking. A 1 mL aliquot was then extracted from the cultures after 2 h and every half hour afterward for 2.5 h for extracellular purine and CFU determination. Samples from purine analyses were centrifuged at 2,000 g and 4°C for 10 min. The supernatant was then filtered (Thermo Fischer TITAN, 0.45 μm, nylon) and metabolite analyses performed as described below.

#### Transepithelial resistance (TEER) measurement

Cells were plated on 0.33 cm^2^ transwell inserts (Corning, 0.4 µm) at 58,000 cells/insert, and TEER readings were measured over 7 days using an epithelial volt-ohm meter (EVOM,^2^ World Precision Instruments). Where indicated, the cells were treated with 1 mm butyrate, hypoxanthine, or both, with the media and treatments refreshed daily.

#### Oxygen consumption analyses

Cells were plated on 0.33 cm^2^ transwell inserts (Corning; 0.4 μm) and grown to confluency and full barrier resistance as indicated by TEER. Cells were then treated with 1 mm butyrate, hypoxanthine, or butyrate and hypoxanthine and oxygen levels monitored overnight using an OxoDish OD24 (PreSens) and SDR SensorDish reader (Presens) at 37°C.

#### E. coli *strains generation*

The *E. coli* strains were generated through incorporation of stop codons through multiplex automated genome engineering (MAGE) using the pORTMAGE-3 plasmid. pORTMAGE-3 was a gift from Csaba Pál. The BW25113 parent strain was transformed with the pORTMAGE-3 plasmid and MAGE recombineering was performed as described previously.^84^ The E. coli ΔrpsL mutagenic oligonucleotide was synthesized with four phosphorothioate linkages at the 5′ terminus.^85^ The Δ*rpsL* R86S mutant was selected through plating on LB agar with kanamycin and streptomycin. The triple mutant (Δ*purR* Δ*gpt* Δ*hpt*) was constructed through multiple cycles of pORTMAGE mutagenesis using a mixture of modified mutagenic oligonucleotides. Validation of the mutants was performed with colony PCR using wildtype- and mutant-specific primers for each target followed by Sanger sequencing of genomic DNA (not shown).^86^ The mutant strains were cured of the pORTMAGE-3 plasmid through growth at 37°C. Verification that the resulting strains had lost the pORTMAGE-3 plasmid was performed through replicate plating on single and dual selection LB agar.

#### HPLC analyses

##### Extracellular fecal metabolites

Fecal pellets were removed from −80°C and dispersed in 350 µL of ice-cold LC-MS grade water. All extractions were performed on ice. The fecal pellet was manually dispersed using a 1 mL pipette tip and then centrifuged at 2,000 g and 4°C for 10 min. The supernatant was then transferred to a new tube, another 350 µL of LC-MS water added to the fecal matter, and the matter resuspended. The extraction process was then repeated two more times, producing a total of 1.05 mL of extract. The extract was then filtered through a 0.2 um nylon filter (Thermo Scientific Titan3, 17 mm) and submitted to metabolite analyses.

##### Colon tissue metabolites

Colon tissue metabolites extracted as previously described with minor variations.^26,27^ All extractions were performed on ice. For metabolite extraction, the tissue was first sonicated 3 × 5 s (BioLogics Inc., 150 V/T Ultrasonic Homogenizer, power output ∼20). The sample was then placed in liquid nitrogen until frozen, removed and thawed, and vortexed for ∼10 s. The sample was centrifuged for 10 min at 18,000 g and 4°C, and the supernatant transferred to a new Eppendorf tube. Another 500 μl of 80% MeOH was then added to the sample, the tissue resuspended, and the extraction process repeated twice. The resulting 1.5 ml of extract was dried via an Eppendorf Vacufuge at room temperature. The dried extract was dissolved in 350 μl of LC-MS water and filtered (Thermo Fischer TITAN, 0.45 μm, nylon) for metabolite analyses.

##### HPLC-DAD

Analyses were performed on an Agilent Technologies 1260 Infinity HPLC using a Phenomenex Luna 5 µm C18(2) column (100 Å, 300 × 4.6 mm) (mobile phase A, 50 mm KH2PO4, 5 mm tetrabutylammonium bisulfate, pH 6.25; mobile phase B, acetonitrile; column temperature, 30°C). Chromatographic separation of the metabolites was performed using a combination of isocratic and gradient methods, including column washing and equilibration periods at the end (0 min: 100% A, 0.3 mL/min; 30 min: 100% A, 0.3 mL/min; 45 min: 100% A, 1 mL/min; 70 min: 92.5% A, 1 mL/min; 130 min: 40% A, 1 mL/min; 175 min: 40% A, 1 mL/min; 185 min: 100% A, 1 mL/min; 215 min: 100% A, 1 mL/min; 216 min: 100% A, 0.3 mL/min; 226 min: 100% A, 0.3 mL/min). The metabolites were detected by absorption at wavelengths of 210, 254, and 280 nm, with their absorbance spectra and retention times verified by co-injection with authentic standards.

##### HPLC-ESI MS

Metabolites were analyzed as previously described with minor variations.^87^ Analyses were performed on an Agilent Technologies 1260 Infinity II LC/MSD iQ with electrospray ionization (ESI) mass detection. Negative ion mass-to-charge ratios (m/z) were scanned from 40 to 200. The metabolite extracts were chromatographed using a Luna 5um HILIC column (200 Å, 250 × 4.6 mm) (mobile phase A: LC-MS grade water; mobile phase B: LC-MS grade acetonitrile; column temperature, 30°C; flow rate, 0.3 mL/min). Chromatographic separation was performed using a combination of isocratic and gradient methods, including column washing and equilibration periods at the end (0 min: 99% A; 20 min: 99% A; 30 min: 10% A; 90 min: 10% A; 100 min: 99% A; 130 min: 99% A). The metabolites were detected by the masses of their negatively charged ions (*M*-1 ± 0.05; butyrate, 87 m/z; acetate, 59 m/z), with their retention times and m/z verified by co-injection with authentic standards.

#### Histological and immunofluorescent analyses

Histological and immunofluorescent analyses were performed as previously described with minor variations.^26,87^ Paraffin embedding of tissue and slide preparation (blank and H&E) was performed by the University of Colorado AMC Department of Pathology histology laboratory. Slides were deparaffinized through a series of washes: xylene, 2 × 3 min; xylene:ethanol:1:1, 3 min; ethanol, 2 × 3 min; 95% ethanol, 3 min; 70% ethanol, 3 min; 50% ethanol, 3 min; rinse in cold tap water.

##### Immunofluorescence

Deparaffinized slides were placed in Tris-EDTA buffer (10 mm Tris base, 1 mm EDTA, 0.05% Tween 20, pH 9.0) for heat-induced epitope retrieval (HIER) (Biocare Medical Decloaking Chamber, DC2008US). HIER was performed at 80°C for 1 h (Ki67 and TIGAR) or 125°C for 30 s (ZO1) and the slides then equilibrated to room temperature. The slides were washed 2 × 5 min in TBS wash buffer (50 mm Tris, 150 mm NaCl, 0.025% Triton X-100, pH 7.6), the tissue permeabilized by immersion in TBS wash buffer containing 0.2% Triton X-100 for 8 min, then the slides washed again 2 × 5 min in TBS wash buffer. The tissue was blocked using 10% normal goat serum and 1% bovine serum albumin (BSA) in Tris buffered saline (TBS, 50 mm Tris, 150 mm NaCL, pH 7.6) for 2 h at room temperature. Primary antibody in TBS containing 1% BSA (Ki67, 1:100; TIGAR, 2.5:1000; ZO1, 1:100) was then added to the tissue and the slides incubated overnight at 4°C. The slides were then washed 2 × 5 min in TBS wash buffer and secondary antibody added (1:500 in 1% BSA TBS) and incubated for 1 h at room temperature. Then, the slides were rinsed 3 × 5 min in TBS, a coverslip added using ProLong Diamond Mountant with DAPI, and the slides cured for 24 h. Fluorescent images of the tissue were taken using a Zeiss AxioCam MRc 5 at 100X or 200X magnification. Multiple, non-overlapping images of each tissue section from each mouse group (*n* = 5 if possible) were taken, and all images were used for quantification. Images were processed and quantitated using the Zeiss Zen2 program. Immunofluorescence was quantified by drawing regions of interest around the epithelium and normalized to DAPI (target/DAPI, fluorescence sum/sum), except for Ki67, where the number of Ki67 positive was counted along with DAPI to determine the fraction of proliferating cells per crypt. Ki67 was procured from Thermo Fischer Scientific (Cat# 14-5698-82), TIGAR from Abcam (Cat# ab62533), and ZO1 from Millipore (Cat# MABT11).

#### Shotgun metagenomic analyses

DNA was isolated from flash-frozen fecal pellets using a QIAamp PowerFecal Pro DNA Kit (QIAGEN Cat# 51804) and further purified via a DNeasy PowerClean Cleanup Kit (QIAGEN Cat# 12877). Shotgun metagenomic sequencing was performed by Novogene (12 Gb raw data per sample).

##### Functional enrichment

Analyses to assess functional enrichments in the microbiota at the genomic were performed using Anvi’o.^40–42^ Genomes were reconstructed from the shotgun metagenomic sequences through assembly using metaSPAdes,^88^ or IDBA-UD^89^ if metaSPAdes exceeded our memory availability, and then resulting contigs binned using MetaBAT2^90^ as a heuristic followed by manual bin refinement iterations to maximize metagenome-assembled genome (MAG) completeness and minimize redundancy. Bins representing MAGs that were > 50% complete and/or >2 M basepairs with < 10% redundancy were used for analyses. The MAGs were then annotated with functions from the KOfam HMM database of KEGG orthologs.^43,44^ Taxonomic estimates of the MAGs were performed in Anvi’o, which currently uses single-copy core genes found in GTDB genomes,^45^ and functional enrichment comparisons were performed amongst the MAGs in groups at the genus taxonomic level (each group compared to all other groups).^46–48^ User-defined metabolic modules used for functional enrichment analyses are available upon request.

#### Bulk RNA sequencing and analysis

Colon tissue was added to TRIzol Reagent (Invitrogen), homogenized, and RNA purified according to manufacturer’s protocol. RNA was then submitted to Novogene for sequencing (9 Gb Raw data per sample). The RNAdetector pipeline was used for data analysis,^91^ which uses Trim Galore for quality trimming (http://www.bioinformatics.babraham.ac.uk/projects/trim_galore/). Alignment and read quantification was executed using STAR^92^ and featureCounts,^93^ respectively, referenced to the murine GENCODE M36 (GRCm39) release. Genes below the median of the overall count distribution were filtered, normalized using edgeR^94^ Trimmed Mean of M-values (TMM), and differentially expressed gene statistics determined by limma.^95^

#### Statistical analyses

Statistical analyses were performed in GraphPad Prism 10 using an unpaired two-tailed parametric t-test with Welch correction for direct comparisons. Statistical differences were considered significant when *p* ≤ 0.05. Suspected outliers were tested using Grubbs’ method with alpha = 0.05. Additional statistical details can be found in the figure legends.

## Supplementary Material

Supplemental Material
